# The tale of the rattle: Using rattle size to understand growth and sexual dimorphism in an insular population of rattlesnakes (*Crotalus oreganus caliginis*)

**DOI:** 10.1002/ece3.70005

**Published:** 2024-07-10

**Authors:** Roman A. Nava, José Jesús Sigala‐Rodríguez, Nathaniel Redetzke, Ivan Villalobos‐Juarez, Cristian Franco‐Servin‐de‐la‐Mora, Ramses Rosales‐García, Rulon W. Clark

**Affiliations:** ^1^ Department of Biology San Diego State University San Diego California USA; ^2^ Moffett Federal Airfield Mountain View California USA; ^3^ Departamento de Biología Universidad Autónoma de Aguascalientes Aguascalientes Mexico; ^4^ Environmental Security ‐ Uplands Management Section Marine Corps Base Camp Pendleton Camp Pendleton California USA; ^5^ Department of Biological Sciences Clemson University Clemson South Carolina USA

**Keywords:** body size, Coronado Island rattlesnake, insular evolution, island dwarfism, Islas Coronado, ontogeny, SSD, Viperidae

## Abstract

Islands have played a key role in our understanding of rapid evolution. A large body of literature has examined morphological changes in response to insularity and isolation, which has yielded useful generalizations about how animals can adapt to live in very small geographic areas. However, understanding the evolution of morphological variation in insular populations often requires detailed data sets on longitudinal patterns of growth and development, and such studies typically necessitate long‐term mark‐recapture on a large sample of individuals. Rattlesnakes provide a unique opportunity to address some of these difficulties because the addition of rattle segments to the rattle string occurs with regular periodicity and their size directly correlates with the body size of the snake at the time of the ecdysis cycle generating the segment. Here, we used a large database of rattle segment sizes recorded from island (Isla Coronado Sur, Baja California, Mexico) and mainland (Camp Pendleton, California, United States) populations of Western Rattlesnakes (*Crotalus oreganus* and *C. o. caliginis*) that separated approximately 10,000 years ago to compare body sizes at different ecdysis cycles, which allowed us to assess differences in growth rates and patterns of sexual size dimorphism. Our results show that rattlesnakes on Isla Coronado Sur appear to be born smaller and grow more slowly than their mainland counterparts, resulting in a “dwarfed” island population. However, despite significant differences in body size, both populations exhibited the same degree of sexual dimorphism. Our study demonstrates the potential to use rattle characteristics to recover detailed estimates of fundamental demographic parameters.

## Graphical abstract

We examine unique rattlesnake rattle characteristics to address some of the shortcomings in long‐term data sets when examining morphological changes in an island and mainland population of rattlesnakes. We used a large database of rattle segment sizes recorded from both island and mainland populations of Western Rattlesnakes (*Crotalus oreganus*) and Coronado Island Rattlesnakes (*Crotalus oreganus caliginis*) to compare approximate body sizes at different ecdysis cycles, which allowed us to assess differences in growth rates and patterns of sexual size dimorphism. Our results show that rattlesnakes in our study island population grow more slowly than their mainland counterparts, resulting in a “dwarfed” island population.

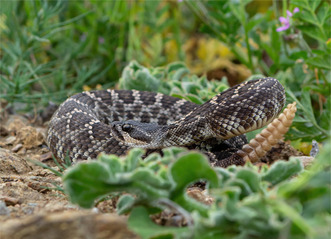

## 1 INTRODUCTION

Islands have played a major role in our understanding of reproductive isolation and speciation (Warren et al., 2015). Terrestrial species isolated on islands are often completely separated from mainland populations and can rapidly diverge in behavior and morphology (Benítez‐López et al., 2021; Meiri et al., 2006; Sacchi et al., 2015). Island populations frequently exhibit dwarfism or gigantism, which has led to ecological rules characterizing body size changes in different taxonomic groups.

Island dwarfism and gigantism have evolved several times in groups of terrestrial vertebrates, and the general “island rule,” first coined by Van Valen states that island populations of normally large animals tend to get smaller, while island populations of normally small animals get larger. Several studies have analyzed the generality of the rule, showing that different taxonomic groups exhibit unique responses that may deviate from Van Valen's expected patterns (Benítez‐López et al., 2021; Lomolino, 1985; Meiri et al., 2006; Van Valen, 1973). In reptiles, the island rule may not hold particularly well for some taxa, likely due to different mechanisms than those driving patterns in other vertebrate groups (Meiri et al., 2006). For example, in comparison with other taxonomic groups, reptiles are often more strongly affected by the seasonality and abundance of resources but still exhibit a general trend of small species undergoing gigantism and large species undergoing dwarfism (Benítez‐López et al., 2021). The most prominent pattern from a meta‐analysis of island lizard populations was that, when compared to mainland populations, island lizards had increased population density and had smaller clutches with larger hatchlings (Novosolov et al., 2013).

For snakes, island populations frequently exhibit either dwarfism or gigantism, depending on typical adult body size (snakes over ~1 m tend toward dwarfism, and under ~1 m toward gigantism) and food resources (snakes on islands with smaller‐bodied prey tend toward dwarfism) (Boback, 2003; Boback & Guyer, 2003; Keogh et al., 2005). Although numerous studies have compared typical or maximum adult body sizes of island and mainland snakes (e.g., Ashton, 2001; Keogh et al., 2005; Vanek & Burke, 2020), the majority of these use a static sample or collection of individuals. Thus, the relative patterns of growth and age at sexual maturity often remain unknown. Because snakes and other reptiles exhibit plastic growth rates, understanding patterns of growth and maturity can be key to a deeper understanding of the mechanisms underlying body size differences associated with island populations.

In addition, it is often unclear if island populations have similar patterns of sexual dimorphism as mainland populations. Larger‐bodied rattlesnakes (i.e., *Crotalus* spp.), barring a handful of exceptions, exhibit male‐biased sexual size dimorphism, with males being approximately 10%–20% larger than females (Klauber, 1956). There are several potential mechanisms that could contribute to this pattern, including sexual selection, physiology, population density, and reproductive stress (Beaupre et al., 1998; Lind et al., 2010; Littleford‐Colquhoun et al., 2019; Taylor & DeNardo, 2005). Comparing patterns of sexual size dimorphism (SSD) between island and mainland individuals can shed light on the relative strength of these mechanisms, as islands often diverge strongly in factors such as predation pressure, population density, and resource limitation. For example, if sexual selection is an important mechanism driving SSD in rattlesnakes, we would expect to see higher SSD in island populations with increased population density, due to the increased numbers of male conspecifics (Stamps et al., 1997). In squamates, some evidence suggests sexual dimorphism is exacerbated in island populations with higher population densities (Bustos Zagal et al., 2014); but in other cases, it could be minimized, as it is in *Crotalus catalinensis* (Martins et al., 2012).

**FIGURE 1:**
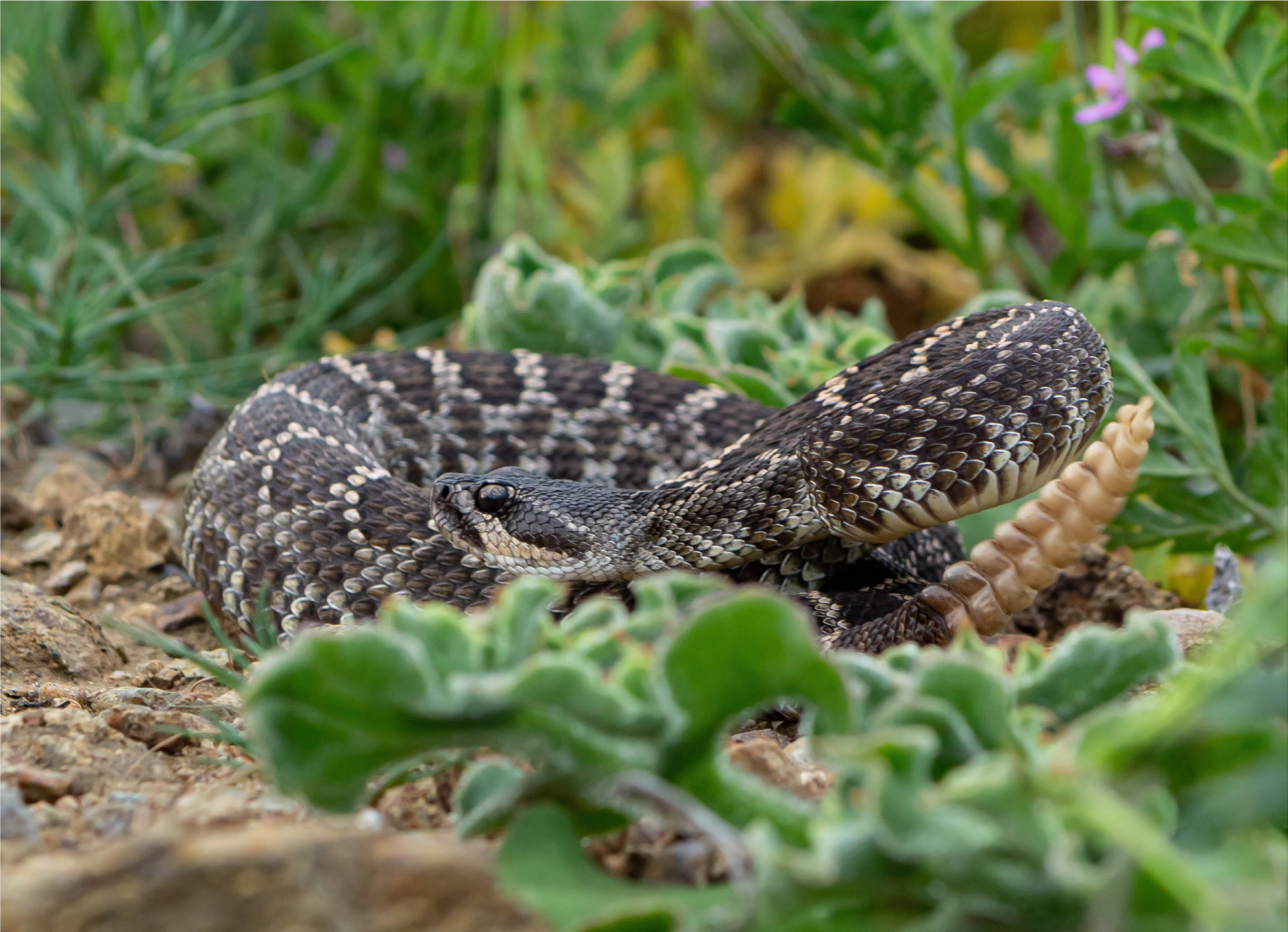
Picture of adult *Crotalus oreganus caliginis*, in situ, demonstrating rattle with broader segments at base, and narrower (older) segments toward the tip of the rattle. Segment width correlates positively with SVL, illustrating the utility of rattle metrics to measure body size and growth rates of a population.

Although a deeper understanding of the ontogeny of body size would help in establishing what ecological and environmental factors may lead to morphological differences on islands, for species on many islands longitudinal data on growth rate are difficult and time‐consuming to obtain due to limited or sporadic access or facilities. However, because of the unique characteristics of the rattle, rattlesnakes (genera *Crotalus* and *Sistrurus*) present an opportunity to quantify variation in body size and growth rate using a static sampling scheme. The rattle of rattlesnakes is a remarkable structure that not only serves to produce an effective aposematic display but also develops in a fashion such that the size of the segments reflects the growth history of the snake bearing the rattle (Klauber, 1956) (Figure 1). The relative width of rattle segments (i.e., measurement of the longer, flatter side of the segment) can be used as a metric of body size and growth, as a new basal segment is added each time the snake sheds its skin (Brown, 1991). The more distal segments thus serve as a record of the relative size of the snake at the time at which the segment was created, and several previous studies have demonstrated the strong correlation (e.g., *r* > .9) between snake body size and segment width (Beaupre et al., 1998; Brown, 1991). Reiserer (2016) provides a comprehensive review of the technique of using rattle characteristics to estimate growth rates.

The Western Rattlesnake (*Crotalus oreganus*) is a large‐bodied species distributed widely across western North America. This species also occurs on two oceanic islands and three freshwater lake islands (Ashton, 2001). Although all five island populations are reported to be dwarfed in body size (Ashton, 2001; Klauber, 1956), no previous studies have taken advantage of the growth rates that can be extracted from rattle characteristics to determine how patterns of growth, sexual maturity, and sexual size dimorphism differ between two closely related but evolutionarily isolated populations. Here, we use measurements of body size and rattle segment width to compare relative growth rates and patterns of sexual size dimorphism in one of these island populations, *C. o. caliginis* (the subspecies of Western rattlesnakes isolated on Isla Coronado Sur) to a nearby mainland population from coastal Southern California (Camp Pendleton) (Figure S1). Based on previously published studies, we hypothesized that *C. o. caliginis* would be dwarfed but would exhibit a greater degree of SSD than *C. oreganus*. We also hypothesized that due to a lack of mammalian prey, *C. o. caliginis* would exhibit rattle characteristics consistent with slower growth rates.

## 2 METHODS

### 2.1 Focal species

Western Rattlesnakes (*C. oreganus*) are distributed throughout western North America in a wide variety of habitats. These ambush predators typically prey on lizards as juveniles and shift to prey on small‐to‐medium‐sized mammals as they mature (Dugan & Hayes, 2012; Sparks et al., 2015). They are active in southern California from late March to November, brumating (hibernating) over the winter. Western Rattlesnakes in southern California typically emerge from brumation at the beginning of March, exhibit spring mating behavior, and are active until late November, which coincides with another brief mating period at the end of summer (Lind et al., 2010). *Crotalus oreganus* is one of the most common large snake species in southern California and likely plays a key role in its ecological community via consumptive and nonconsumptive impacts on small mammal and lizard populations (Bouskila, 1995; Brown & Kotler, 2004). Compared to other similar‐sized vertebrate predators, most pitvipers can live in high densities or in small patches of land with a limited resource base due to low energy requirements relative to other similar‐sized vertebrates like mammals, birds, and lizards (Nowak et al., 2008).

In addition to their mainland distribution in Southern California, a dwarfed population of *C. oreganus* persists on Isla Coronado Sur of the Islas Coronado, Baja California, Mexico. This population was elevated to a separate subspecies, *C. o. caliginis*, by Klauber (1949). There have been numerous attempts to clarify the *Crotalus* taxonomy, with mixed results. Taxonomic designations are further complicated due to disagreements over what constitutes a species and the convoluted history of this clade (Davis et al., 2016). Here, we follow the general consensus that *C. o. caliginis* represents a subspecies of *C. oreganus* rather than a separate species, although future research may alter our understanding of this taxonomy. *Crotalus oreganus caliginis* occurs in very high densities on Isla Coronado Sur (Grismer, 2002), which is the largest of the Islas Coronado, a chain of four small islands about 16 km off the coast of Tijuana, Baja California, Mexico. While mainland *C. oreganus* regularly reaches over 1 m in length, *C. o. caliginis* specimens analyzed by Klauber (1956) were approximately 52 cm snout‐vent length (SVL) as reproductively mature adults.

### 2.2 Study sites, Islas Coronado

The Islas Coronado house several large seabird colonies and marine mammals. Ten species of reptiles and amphibians occur across the island chain (Grismer, 2002; Kuper & Hart, 1978). Most of the chain's biodiversity is present on Isla Coronado Sur, the largest island at 1.8 km^2^ (the second largest island, Isla Coronado Norte, is less than 1 km^2^) but most of this area is inaccessible to many terrestrial animals and birds because it consists of cliff‐like slopes.

Previous geological surveys found that the island chain split from the mainland between 9000 and 10,000 years ago (McCain et al., 2019). Thus, the island's flora and fauna are still relatively similar to the mainland. Public access is restricted, and there are usually only four Mexican Navy personnel on the island at any given time. The island has effectively operated as a nature preserve since the 1930s, when the Mexican government seized control of the islands from private developers (Kuper & Hart, 1978). In 2016, the Mexican government formally protected the islands under the newly established Pacific Islands Biosphere Reserve.

### 2.3 Study sites, Camp Pendleton

Our mainland sample site for *C. oreganus* was Marine Corps Base Camp Pendleton (Camp Pendleton), a large land area owned and operated by the United States Marine Corps in San Diego County, California, United States. It spans roughly 27 km of coastline and, at 505 km^2^, is by far the largest area of relatively undeveloped coastal habitat in Southern California. Human housing and development are restricted to small parts of the southern region of the base, accounting for 8400 acres, or roughly 6.7% of the total base size. Most of the land area on the base is a mixture of nonnative grasslands or undeveloped chaparral and coastal sage scrub habitat, which supports large populations of native flora and fauna, including *C. oreganus*.

### 2.4 Body size and rattle characteristics

Data on body size and rattle characteristics were collected from snakes captured and released on Isla Coronado Sur (*C. o. caliginis*) and Camp Pendleton in San Diego County (*C. oreganus*) between 2015 and 2020. Snakes were opportunistically collected via visual encounter surveys across accessible (i.e., relatively flat terrain) habitats on Isla Coronado Sur. Snakes from Camp Pendleton were collected via a combination of targeted visual encounter surveys and opportunistic encounters while conducting a long‐term radio telemetry study examining the impacts of short‐distance translocations as a mitigation strategy. All snakes were processed within a few days of capture and were released at the capture site, or within 1 km of the capture site in cases on Camp Pendleton where snakes had to be relocated by base officials to reduce human–wildlife conflict.

We measured several morphological traits, including SVL. We estimated SVL to the nearest centimeter using the tubing method in order to safely handle individuals and minimize handling stress. Briefly, this method involves coaxing the snake into a clear plastic tube for safe restraint and using a flexible measuring tape run down the middle of the dorsum of the snake from the tip of the snout to the cloaca. We also measured weight, tail length (length from cloaca to the base of the rattle), sex via probing of hemipene pocket, and the number and width of rattle segments. We used calipers to measure each segment to the nearest 0.1 mm and noted if the individual had a broken or intact rattle. Broken rattles are missing one or more segments at the end of the rattle. Intact rattles still have the characteristic rounded “button” (the first segment of a newborn rattlesnake) intact. Snakes were injected subdermally with a PIT tag for reidentification in the case of recaptures.

We used our sample of snakes with complete rattles to produce a comparison of rattle segment widths between *C. oreganus* and *C. o. caliginis*. Counting and estimating rattle segment numbers allowed us to compare the two populations at the same approximate age, as rattlesnakes shed their skin in nature with regular periodicity, producing a new segment with each shed (ecdysis) cycle (Brown, 1991; Diller & Wallace, 2002). As an additional measure of body size difference between sites for juveniles, we statistically compared the measured SVL of live snakes in our samples that had three segments (button plus three more segments) on the rattle, as this was the smallest segment value for which we had a large enough sample of measured snakes for comparison.

We also compared the relative number of individuals in each population that had reached asymptotic growth (i.e., the relative proportion of older adults present). For this comparison, we categorized snakes with three or more adjacent segments that were all within 1 mm width of each other as achieving asymptotic growth. For individuals in this condition, we counted the number of similar‐sized segments inclusive of these first three as a metric of the minimum number of shed cycles that an individual had undergone since it reached adult body size.

We quantified sexual size dimorphism in both populations independently by comparing adult SVL for adult males and females. Because we could not collect data on sexual maturity of individuals without sacrificing them, for this comparison we used Klabuer's (1956) smallest gravid female length value (table 4.1) minus average female tail length (table 4.3) for *C. o. caliginis* and *C. o. helleri* to categorize mainland rattlesnakes larger than 589 mm SVL as adults and island rattlesnakes larger than 522 mm SVL as adults (Diller & Wallace, 2002; Klauber, 1956). Note that this estimate of adult size is conservative for island snakes (i.e., they probably reach sexual maturity at smaller body sizes than this), as it is likely Klauber examined the reproductive condition of many fewer females from this isolated and difficult to access location than from mainland Southern California.

### 2.5 Statistical analyses

We used Gaussian models for continuous numeric data, such as SVL, and analyzed these data for normality and equal variances using the Shapiro–Wilk and *F* tests. For rattle segments, starting with the natal segment (the first segment of a rattlesnake's rattle, the uniquely shaped button), we computed a population level mean, standard deviation, and a 95% confidence interval to compare segment sizes and SVL between populations and sexes with *t*‐tests. Significance was determined with alpha <.05. When normality and equal variance assumptions were not met, we used a nonparametric Wilcoxon test for analysis. We used a Poisson test for our count data, which was limited to counting the number of rattle segments present when the rattle had asymptoted. Statistical analyses were conducted using R version 1.3.1093. All values are reported as mean ± standard deviation.

## 3 RESULTS

### 3.1 Patterns of SSD in adults

**FIGURE 2:**
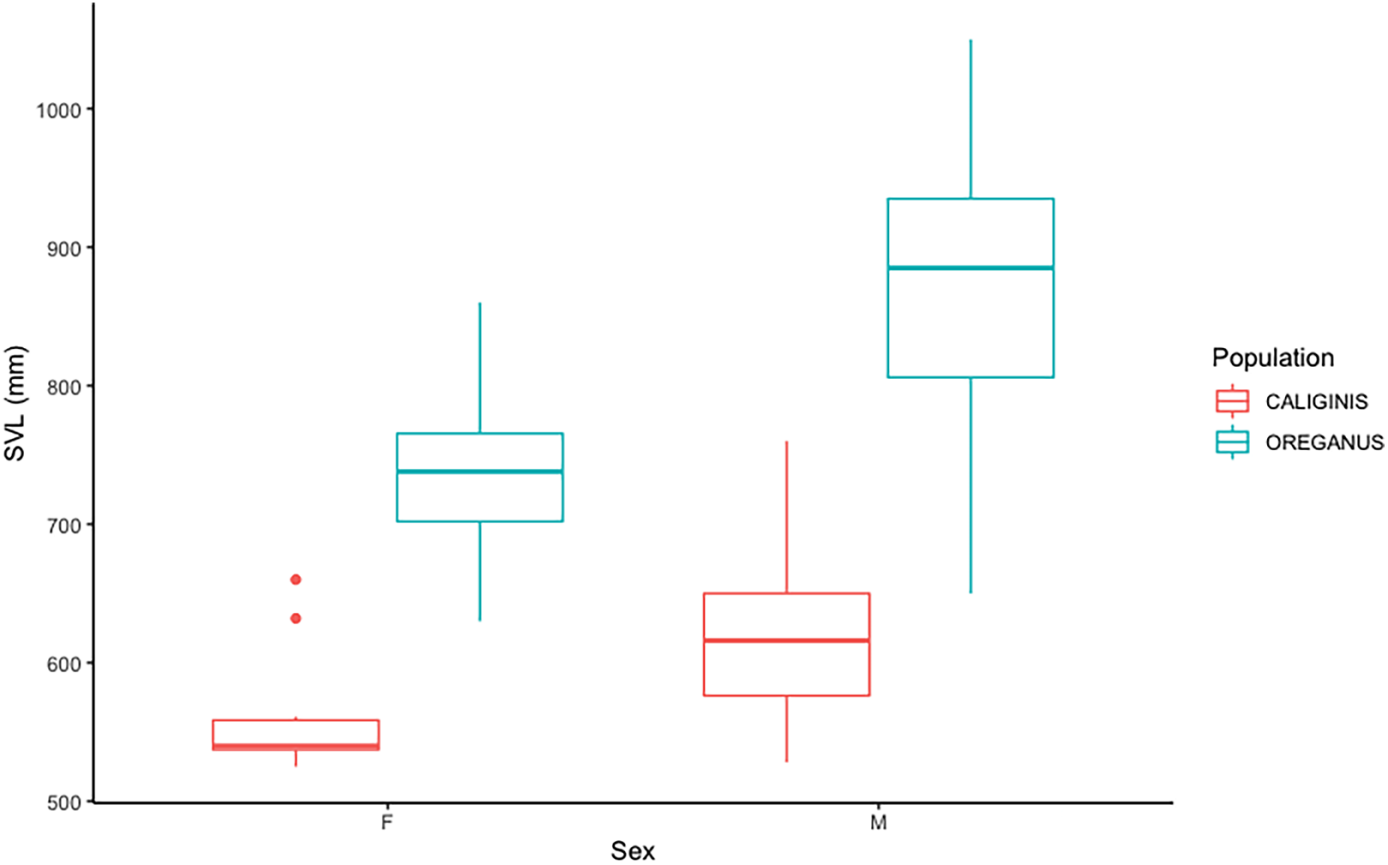
Differences in body size between males, females, and all adults. *Crotalus oreganus* adults of both sexes are larger than *Crotalus oreganus caliginis* adults, and there is a similar degree of male‐biased body size dimorphism in both populations.

We quantified sexual size dimorphism in both populations independently by comparing adult SVL for males and females. Adult males in both populations were significantly larger than adult females. Adult *C. o. caliginis* males were 619 ± 62 mm in SVL compared to 556 ± 40 mm for females (*n* = 54 males, 14 females; *W* = 140.5; *p* < .004). On the mainland, adult *C. oreganus* males *were* 865 ± 99 mm in SVL, whereas females were 737 ± 62 mm in SVL (*n* = 109 males, 30 females; *W* = 504; *p* < .001) (Figure 2). The relative difference in adult body size on the island and mainland population was essentially identical, with *C. o. caliginis* females at 89% of male body size, versus 85% for *C. oreganus*.

### 3.2 Comparisons of body size

Our comparison between snakes we measured directly that had only three rattle segments showed that mainland snakes were already significantly longer (20%) than island snakes by their third ecdysis cycle (mainland snakes = 54, island snakes = 9; *f* = 30.7; *p* < .01). As with previous studies, we also found that mainland adults were approximately 39% larger than adults on Isla Coronado Sur (840 ± 106 mm and 606 ± 63 mm, respectively; mainland snakes = 143, island snakes = 68,  *W* = 286.5; *p* < .001) (Figure 2). We also compared adult mainland males and females to their island counterparts, and mainland adult snakes were significantly larger for both males and females (Figure 2). Females on the mainland are on average 32% larger than island females (737 ± 62 mm and 556 ± 40 mm, *n* = 30 mainland, 14 islands; *W* = 7; *p* < .001). Mainland males were 40% larger than island males on average (865 ± 99 mm and 619 ± 62 mm, *n* = 109 mainland snakes, 54 island snakes; *W* = 127; *p* < .001).

### 3.3 Comparisons of rattle segments

**FIGURE 3:**
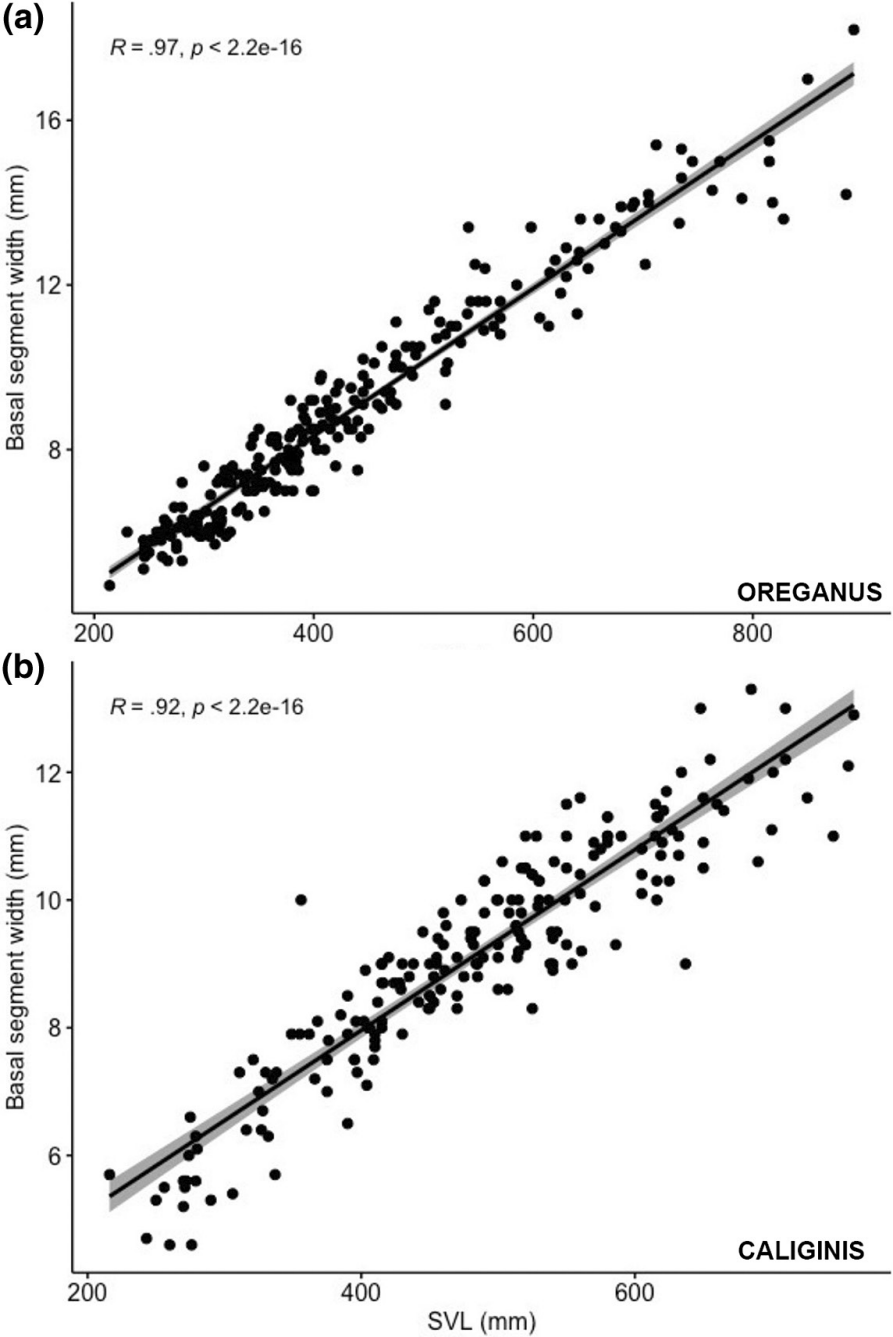
(a) Correlation between basal segment width and snout‐vent length in *Crotalus oreganus* (*n* = 241) and (b) and *Crotalus oreganus caliginis* (*n* = 199).

**FIGURE 4:**
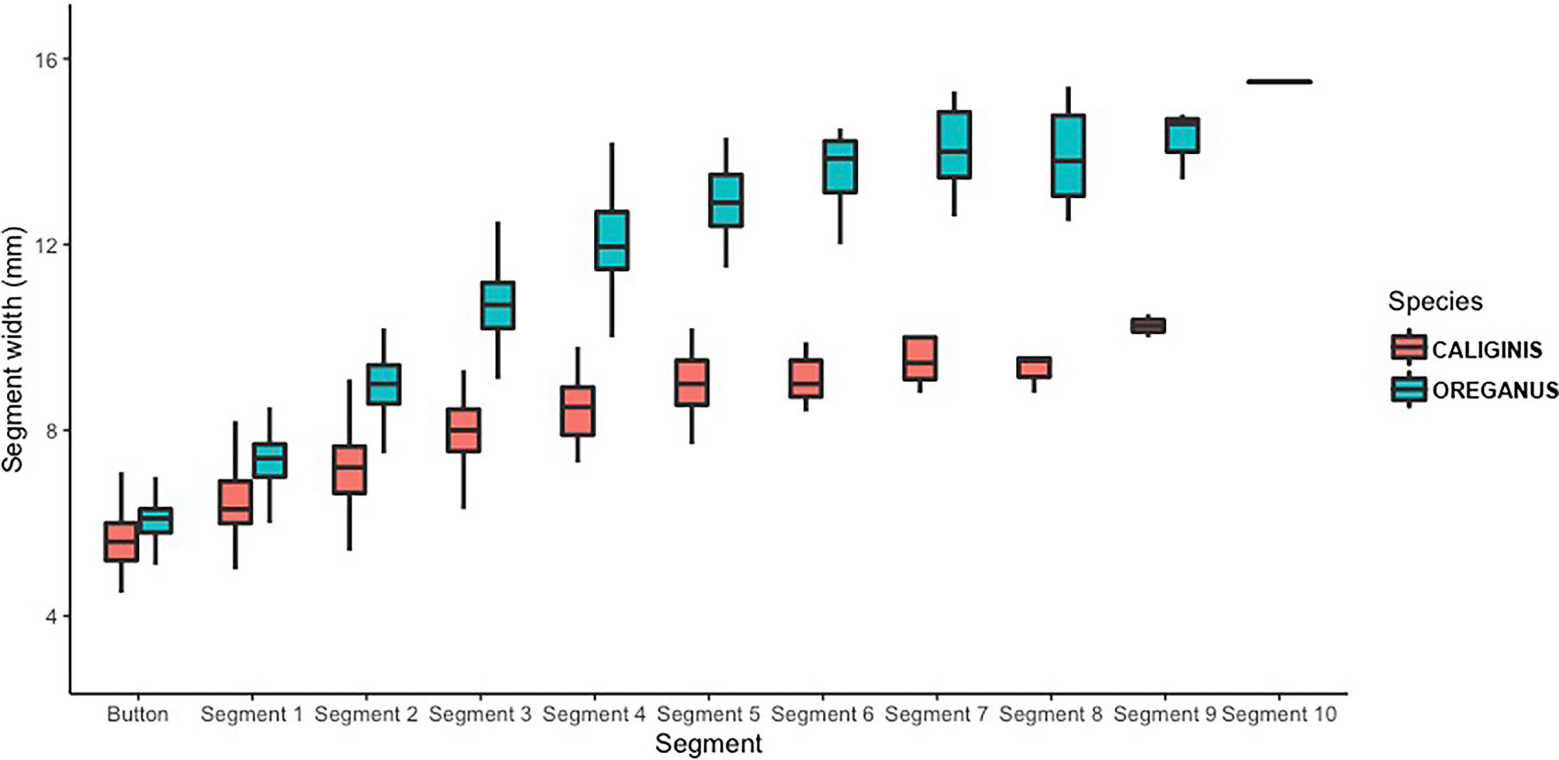
Differences in rattle segment width between *Crotalus oreganus* and *Crotalus oreganus caliginis*. Although the segments are most similar at birth (buttons), *C. oreganus* natal segments (button) are significantly wider, and that difference is exacerbated as segments are added; thus, *C. o. caliginis* rattle segment size asymptotes while snakes are smaller.

**FIGURE 5:**
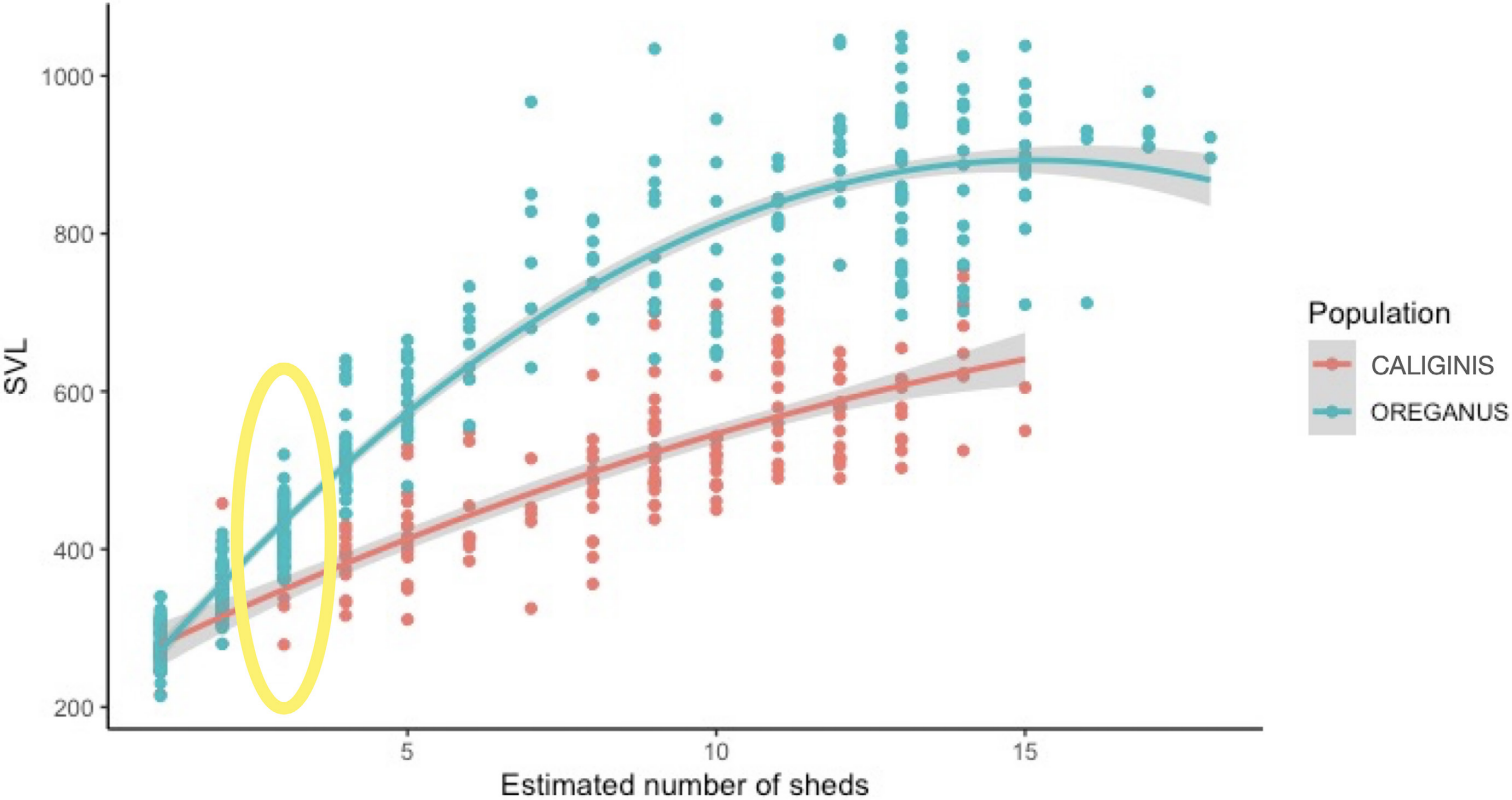
Comparison of snake body sizes at different ecdysis cycles for *Crotalus oreganus* and *Crotalus oreganus caliginis*. *Crotalus oreganus caliginis* is smaller at birth than *C. oreganus* and asymptotes at a smaller body size. Curved line is a least squares line with a 95% confidence interval. The yellow circle at shed three emphasizes the body size discrepancy in *C. oreganus* and *C. o. caliginis*. At this point, *C. oreganus* is already significantly larger than *C. o. caliginis*.

**FIGURE 6:**
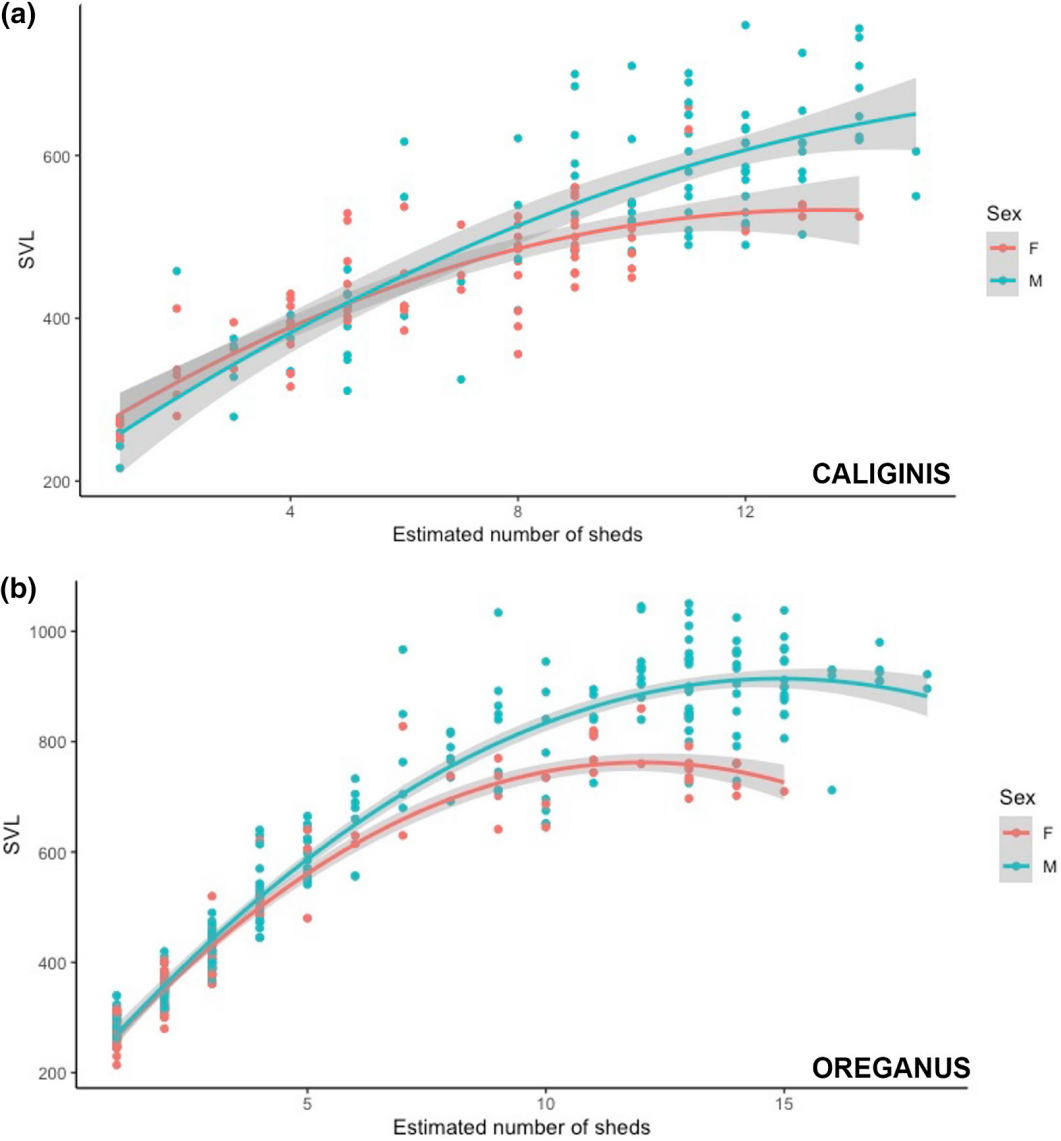
(a) Growth rate of males and females in *Crotalus oreganus caliginis* and (b) *Crotalus oreganus* based on body sizes of individuals measured at different ecdysis cycles (rattle segments). Females stop growing earlier than males and at a smaller size. Curved line is a least squares line with a 95% confidence interval.

As with previous studies, we found a high degree of correlation between basal segment size and body size in both populations (mainland snakes = 241, island snakes = 199; *R* = .97 and .92; *p* < .01) (Figure 3). When comparing chronological rattle segments between sites, we found that mainland and island snakes exhibited different widths for all segments. Although the natal segment width was the most similar rattle segment between the populations, a statistical comparison of the natal segment width showed that mainland snakes have a significantly larger natal segment (*W* = 4293.5; *p* < .001; *n* = 70 island natal segments, 237 mainland natal segments), and that difference increases with each ecdysis cycle (Figure 4). A plot of the relationship between body size (SVL) and ecdysis cycle for both populations also shows that island and mainland snakes are most similar in size at birth, but island snakes grow more slowly and reach asymptotic growth at a smaller body size than mainland snakes (Figure 5). To visualize adult body size and sexual size dimorphism, we plotted the relationship between male and female SVL as a function of rattle size for both populations. These plots revealed that the pattern is similar between populations, with males and females having overlapping growth curves until ecdysis cycle 6 or 7, at which point females in both populations begin to grow more slowly than males (Figure 6a, *C. o. caliginis*; Figure 6b, *C. oreganus*).

Our comparison of adults that had reached asymptotic growth (i.e., had at least three contiguous segments within 1 mm of size) showed that a significantly larger proportion of snakes on the island met these criteria; 110 out of 134 (82%) island adults and 123 out of 172 (71%) mainland adults had achieved asymptotic growth (*χ* ^2^ = 5.1; *p* = .02). Adult mainland snakes that had achieved asymptotic growth had an average of 3.5 rattle segments present after achieving asymptotic growth, whereas island snakes in this condition have 3.6 segments, a difference that was not significant (*n* = 121 island snakes, 123 mainland snakes; *p* = .78, df = 243, AIC = 857.55).

## 4 DISCUSSION

Based on previous studies of rattle characteristics, growth rates, and sexual size dimorphism in the genus *Crotalus*, we hypothesized that *C. o. caliginis* would (1) exhibit reduced body size for both sexes, (2) show an increased degree of male‐biased sexual size dimorphism, and (3) have reduced growth rates. Our findings support our first hypothesis, but we found similar levels of SSD between the populations. We also found support for our third hypothesis, demonstrating island individuals exhibited smaller body sizes at each ecdysis cycle, with the difference becoming greater over time.

More specifically, our analysis of rattle widths indicated that individuals on islands are most similar in size to mainland individuals at birth, as this is the ecdysis cycle that shows the most similarity in segment width, which correlates strongly with body size (Reiserer, 2016). The initial difference in segment width grows more pronounced with additional ecdysis cycles (Figure 4, Tables 1 and 2), indicating that *C. o. caliginis* do not grow as quickly and maintain smaller body sizes at each shed event. Our comparisons of sexes between and within the sites also showed that the island and mainland snakes exhibit broadly similar patterns of sexual size dimorphism. Female adults are approximately 85%–90% SVL of males in both populations and females in both populations exhibited growth patterns that were similar to sympatric males until around the sixth or seventh ecdysis cycle, at which point female growth slowed, whereas males continued to get larger—thus leading to the pattern of male‐biased sexual size dimorphism (Figures 5 and 6). Although there was a significantly larger proportion of island snakes that had reached asymptotic growth compared to mainland snakes (82% vs. 71%), the number of segments present on the rattle string after asymptotic growth was similar between the sites (3.6 vs. 3.5), indicating that the overall demographic structure between the two sites is not drastically different; that is, both sites have a similar proportion of older adult individuals.

**TABLE 1:** Summary of rattle metrics for *Crotalus oreganus* and *Crotalus oreganus caliginis.* S1 = Segment 1, S2 = Segment 2, etc. *C. oreganus* is on the top portion and *C. o. caliginis* follows.

	Button	S1	S2	S3	S4	S5	S6	S7	S8	S9	S10
OREGANUS											
Avg	6.07	7.38	8.98	10.72	12.03	12.9	13.6	13.98	13.9	14.27	15.5
*n*	237	183	128	73	44	25	16	12	6	3	1
SD	0.39	0.59	0.64	0.73	0.91	0.82	0.77	0.92	1.15	0.76	NA
95% CI	0.05	0.09	0.11	0.17	0.27	0.32	0.38	0.52	0.92	0.86	NA
CALIGINIS											
Avg	5.63	6.44	7.22	7.96	8.29	9	9.06	9.46	9.3	10.25	NA
*n*	70	57	51	43	32	18	12	8	3	2	NA
SD	0.67	0.78	0.84	0.79	1.53	0.72	0.47	0.51	0.44	0.35	NA
95% CI	0.16	0.2	0.23	0.24	0.27	0.34	0.27	0.36	0.49	0.49	NA

**TABLE 2:** Percent changes in segment size between each respective segment. B = natal segment, S1 = Segment 1, S2 = Segment 2, etc. *Crotalus oreganus* is on the top row and *Crotalus oreganus caliginis* follows.

	B–S1	S2–S3	S3–S4	S4–S5	S5–S6	S6–S7	S7–S8	S8–S9	S9–S10
Oreganus	20	16.2	12.5	10	8.8	8	7.3	7.3	7.8
Caliginis	14.4	12.2	10.1	4.2	8.5	0.7	4.5	−1.7	0.2

### 4.1 Ecological comparisons within and between sites

Due to their isolation from mainland populations and differing selective pressures (changes in predation, resource availability, habitat type, etc.), island populations tend to diverge relatively rapidly from mainland populations (Eloy de Amorim et al., 2017; Keogh et al., 2005). Differences in behavior, genetic structure, and morphology are common when comparing between sites, and these differences often lead to island speciation. Dwarfism and gigantism are both common morphological differences in terrestrial vertebrates isolated on islands (Lomolino, 1985). This pattern is generally true in reptiles, with small‐bodied reptiles undergoing gigantism and large‐bodied species experiencing dwarfism (Benítez‐López et al., 2021). Unlike mammals, however, island productivity and seasonality also shape body size in reptiles, with large‐bodied reptiles getting smaller on islands with low productivity and high seasonality (Benítez‐López et al., 2021). Based on previous research (Ashton, 2001; Klauber, 1956), we expected to find a pattern of dwarfism for *C. o. caliginis*, and we did indeed find that *C. o. caliginis* was approximately 65% smaller in SVL than mainland *C. oreganus*.

Even though *C. o. caliginis* individuals are smaller than their mainland relatives, they appear to occur at much higher densities (Grismer, 2002; Klauber, 1956; R Nava, R Clark, J Rodriguez, RN, RWC, JSR, personal observation). Intraspecific density is a potential mechanism for dwarfism in island populations, as the increased competition for resources can lead to smaller body sizes (Lomolino, 1985). Although we were not able to access the island frequently enough to confidently estimate population size from mark‐recapture data, qualitatively rattlesnakes were found there at much higher densities than the mainland. We marked ~230 individual *C. caliginis* over the course of 5 years, but only ever recaptured 10 individuals in total; thus, even if our 230 unique individuals represented close to 50% of the total population (which would be an extremely high estimate given our very low recapture rate), the density of snakes on the island would be in the range of 500 snakes/km^2^ at a minimum. The actual population density could be much higher. This is consistent with other qualitative reports (Grismer, 2002; Klauber, 1956) and much higher than most mainland populations of other *Crotalus*, which are typically estimated at around 50–100 snakes/km^2^ (Diller & Wallace, 2002; Kirk et al., 2021; Maida et al., 2017). However, a density of ~500 snakes/km^2^ is similar to densities observed in other island populations of vipers (Almeida‐Santos, 2005; Shine & Sun, 2002; Wen et al., 2021). The reasons for this increased density are likely idiosyncratic across islands, but predator release, lack of interspecific competition, richer resource availability, or higher productivity levels could all contribute to this difference.

Although it is unclear if Isla Coronado Sur has higher primary productivity than mainland habitats, it is possible that productivity may not be as consistent as on the mainland; more data are needed to quantify these qualitative assessments. Further research could investigate the relative availability and energetic quality of prey in islands compared to mainland habitats. Hypothetically, in the absence of predation pressure, *C. o. caliginis* individuals could get larger via predator release (Meik et al., 2010). However, there may be a density‐dependent effect keeping the population large, but the snakes physically small, such as diet quality (Forsman, 1991; Forsman & Lindell, 1996). There is evidence for an optimal body size in viperids, largely regulated by prey size (Boback & Guyer, 2003); that is, viperids only need to be big enough to consume the most abundant or efficiently captured prey in their environment. Larger *Crotalus* species can prey on fully grown cottontail rabbits, and even larger birds like young turkeys (Klauber, 1956). There can even be variation among different populations of the same species—coastal *Crotalus ruber* populations eat larger prey items than inland *C. ruber* populations and are hypothesized to be larger in average body size as a result (Dugan & Hayes, 2012).

Mainland *C. oreganus* frequently prey on mice, ground squirrels and woodrats (Klauber, 1956) in the range of 10–500 g. There are no comparably sized mammals on the Islas Coronado. *Crotalus oreganus caliginis* instead preys almost exclusively on small‐to‐medium‐sized lizards (5–30 g; although deer mice (*Peromyscus maniculatus*) was reported to occur on the island, it is unclear if a viable population of mice actually persists outside of human‐inhabited areas). Larger snakes can eat larger prey, and in the absence of large prey, large body size may be suboptimal because a snake would need to feed more often (eat greater numbers of smaller prey) to sustain itself. There are several published examples supportive of this hypothesis. The body size of tiger snakes (*Notechis* spp.) on Australian islands is highly correlated with available prey size, with dwarfism on islands with only small prey available, and gigantism found on islands with seasonably abundant larger prey (Keogh et al., 2005). An island population of Brazilian lanceheads that feeds mainly on small invertebrates is dwarfed relative to the mainland population, which feeds primarily on vertebrates (Barbo et al., 2012; Marques et al., 2002). An island population of cottonmouths (*Agkistrodon piscivorous*) in the Florida Keys exhibits no change in body size relative to the mainland and snakes there feed on relatively large fish that are dropped by nesting egrets (McCue & Lillywhite, 2002; Wharton, 1966).

However, another possible explanation for the reduced body size in Isla Coronado Sur is that there is a high turnover rate in the population (out of the nearly 230 sampled *C. o. caliginis* individuals, we had only 10 recaptures). Early senescence and low diet quality have been proposed as possible explanations for *C. cerastes*' diminutive stature (Reiserer, 2016). Early senescence could indicate that individuals do not live very long and that the population has a high growth rate. Our data do not actually indicate that early senescence is occurring on Isla Coronado Sur because adult *C. o. caliginis* snakes did not have fewer fully asymptotic rattle segments compared to adult *C. oreganus*; however, further research would be necessary to more quantitatively estimate demographic parameters in the island population, such as survival and reproductive rates.

### 4.2 Growth rates

Rattlesnakes technically have indeterminate growth (Boback & Guyer, 2003; Shine, 1987), but their size at adulthood (when growth slows dramatically) is limited by extrinsic factors such as resource availability, resource quality, and predation pressure. Based on our results and data from Klauber (1956), the island and mainland populations are most similar in size at birth (average size at birth for *C. o. caliginis* 190 and 275 mm for *C. o. helleri*; Klauber, 1956), but then start to diverge, due to island rattlesnakes growing more slowly between ecdysis cycles. By shed cycle 3 (the first size class for which we had collected sufficient numbers of live snakes for measuring SVL), Coronado Island snakes are on average 520 mm in SVL versus 580 mm for mainland snakes. After approximately 5–7 shed cycles, *C. o. caliginis* individuals appear to asymptote and nearly stop growing, with a few individual exceptions. The average 7‐shed *C. o. caliginis* is 532 mm long, whereas the average 7‐shed *C. oreganus* is over 800 mm long. Thus, *C. o. caliginis* apparently grows at a slower rate and asymptotes at a smaller body size. The difference in growth between segments is also larger in *C. oreganus* than in *C. o. caliginis*. The width of juvenile *C. oreganus* segments increases between 8% and 20% per ecdysis cycle, while *C. caliginis* exhibits a 1%–14% size increase between adjacent segments (Table 2). Both populations asymptote at roughly the same number of sheds, however, indicating a potentially conserved relationship between ecdysis and maturation. It should be noted that our discussion regarding growth and ecdysis cycles assumes that island rattlesnakes undergo ecdysis at a roughly similar rate to mainland snakes, an assumption we were not able to test. However, studies on various species of *Crotalus* reveal a largely similar periodicity of ecdysis across populations and species, with juveniles shedding slightly more frequently than adults, and adults of both sexes shedding one or two times per year (reviewed in Carnes‐Mason & Beaupre, 2023).

Broadly, the demographics of the island and mainland populations seem similar, so far as they can be estimated from rattle characteristics. Both populations had a high proportion of individuals that had reached asymptotic growth, although a slightly larger proportion of snakes on the island were in this condition. We sampled no *C. o. caliginis* individuals with more than 10 rattle segments (minimum estimated number of segments) during our study, while we had a few *C. oreganus* individuals that to the best of our estimation had at least 13 segments, but both populations could have had individuals with many more ecdysis cycles, as both contained individuals with rattle strings that demonstrated no tapering of segment width. Tapering rattles (narrower segments toward the tip) indicate periods of rapid growth, typically during an individual's first few years of life (Table 2).

### 4.3 Sexual dimorphism

Previous research indicated that the degree of sexual size dimorphism usually increases in isolation from other populations (Bustos Zagal et al., 2014). Our results suggest that this is not the case with *C. o. caliginis*, as males are 11% larger than females on South Coronado Island and 17% larger on the mainland. These rates are on the higher end of dimorphism in snakes (de Almeida‐Santos, 2005; Shine, 1994), but still within the expected range.

Although it has been historically assumed that SSD in rattlesnakes is driven by intra‐ or intersexual selection, several researchers have contested this view. Patterns of SSD have been shown to be plastic, with little or no SSD present in captive populations (Taylor & DeNardo, 2005). This suggests that size is shaped by extrinsic factors, such as resource availability (Taylor & DeNardo, 2005). There is also a strong pattern of lack of female choice in rattlesnake mating systems (Levine et al., 2020) and reptiles in general (Uller & Olsson, 2008) indicating that intersexual selection alone is unlikely to lead to SSD.

In terms of intrasexual selection, male–male combat is common during the mating season for most rattlesnakes, and thus could be a selective force for increased male body size, as empirical studies in viperids indicate larger males generally win combats (Gillingham et al., 1983). This effect could be particularly strong when male population density is high, thus increasing the occurrence of male–male encounters. A male that defeats another male could hypothetically have increased access to reproductive females, but male combat and mating/courtship behaviors are still rarely observed in rattlesnakes, and studies on *C. atrox* (another rattlesnake species where males are significantly larger than females) found no evidence for sexual selection operating on male body size (Clark et al., 2014; Levine et al., 2020).

Some researchers have proposed that SSD in rattlesnakes is more strongly driven by selection for optimal female growth related to reproduction (Nowak et al., 2008). Female snakes invest heavily in reproductive effort, often losing 40%–50% of their body mass following live birth (Beaupre, 2002). Following birth, growth is minimized to restore fat reserves, and additional energy acquisition is continually shunted toward reproduction overgrowth. Since males do not experience similar levels of weight loss associated with reproduction, their growth may be more optimized to take advantage of available prey resources, leading to larger body sizes in populations with large‐bodied prey availability, while female body size results from the balance in resource allocation between growth and reproductive effort. Under this hypothesis, differences in SSD across species and populations would be more related to variation in female reproductive cycles or effort, and less related to population density and the frequency of male–male combat. These hypotheses are also not mutually exclusive, and some combination of selective pressures for intrasexual combat and optimal body size could result in the patterns of SSD seen in pitvipers.

## 5 CONCLUSION

Our analysis of *C. oreganus* and *C. o. caliginis* demonstrated these species differ in size and growth rate but not in the observed degree of SSD. Island dwarfism occurs in this species because they are born smaller, grow slower, and stop growing at a smaller body size. Our study further underscores the utility of using rattle characteristics to estimate key population parameters, as we would not have been able to estimate body sizes at different life stages or patterns of growth and maturation with static samples of body size alone. We would encourage future studies to investigate the relationship between resource availability, prey size, and body size in viperids, as well as the demographic implications of these patterns and the mechanisms that may lead to differences in body size between males and females.

## AUTHOR CONTRIBUTIONS

**Roman A. Nava:** Conceptualization (equal); data curation (equal); formal analysis (equal); investigation (equal); methodology (equal); project administration (equal); validation (equal); visualization (equal); writing – original draft (equal); writing – review and editing (equal). **José Jesús Sigala‐Rodríguez:** Conceptualization (equal); data curation (equal); investigation (equal); project administration (equal); resources (equal); supervision (equal); writing – review and editing (equal). **Nathaniel Redetzke:** Investigation (equal); writing – review and editing (equal). **Ivan Villalobos‐Juarez:** Investigation (equal); writing – review and editing (equal). **Cristian Franco‐Servin‐de‐la‐Mora:** Investigation (equal); writing – review and editing (equal). **Ramses Rosales‐García:** Investigation (equal); writing – review and editing (equal). **Rulon W. Clark:** Conceptualization (equal); funding acquisition (equal); investigation (equal); methodology (equal); project administration (equal); resources (equal); supervision (equal); writing – review and editing (equal).

## CONFLICT OF INTEREST STATEMENT

All authors have no conflict of interest or competing interest.

## Supporting information


Figure S1.

## Data Availability

All data used in the analysis are available as supplementary data set. https://doi.org/10.6084/m9.figshare.24932895.v1.
