# Supplementary figures and images for: The tale of the rattle: Using rattle size to understand growth and sexual dimorphism in an insular population of rattlesnakes (Crotalus oreganus caliginis)
Source: Ecol Evol. 2024 Jul 10;14(7):e70005. doi: 10.1002/ece3.70005 (PMC11236481; doi:10.1002/ece3.70005)

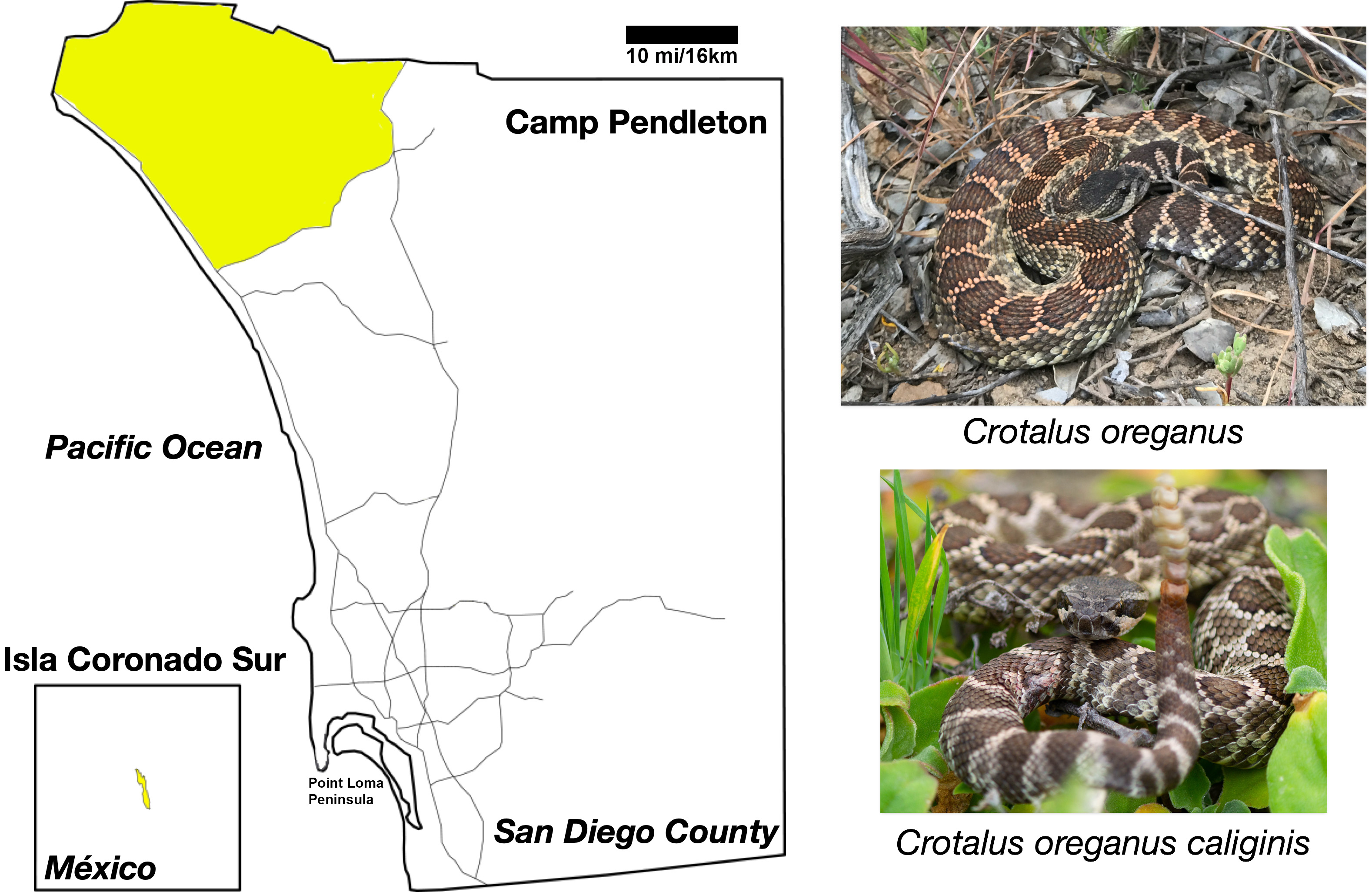

Supplement: Supplementary file 1 — Figure S1. [file ECE3-14-e70005-s001.jpg]
